# Research progress of natural products and their derivatives against Alzheimer’s disease

**DOI:** 10.1080/14756366.2023.2171026

**Published:** 2023-02-20

**Authors:** Jin-Ying Liu, Hong-Yan Guo, Zhe-Shan Quan, Qing-Kun Shen, Hong Cui, Xiaoting Li

**Affiliations:** a Key Laboratory of Natural Medicines of the Changbai Mountain, Ministry of Education, College of Pharmacy, Yanbian University, Yanji, Jilin, China; b Center of Medical Functional Experiment, Yanbian University College of Medicine, Yanji, China

**Keywords:** Natural product, Alzheimer’s disease, cholinease inhibitor

## Abstract

Alzheimer’s disease (AD), a persistent neurological dysfunction, has an increasing prevalence with the aging of the world and seriously threatens the health of the elderly. Although there is currently no effective treatment for AD, researchers have not given up, and are committed to exploring the pathogenesis of AD and possible therapeutic drugs. Natural products have attracted considerable attention owing to their unique advantages. One molecule can interact with multiple AD-related targets, thus having the potential to be developed in a multi-target drug. In addition, they are amenable to structural modifications to increase interaction and decrease toxicity. Therefore, natural products and their derivatives that ameliorate pathological changes in AD should be intensively and extensively studied. This review mainly presents research on natural products and their derivatives for the treatment of AD.

## Introduction

Alzheimer’s disease is an insidious and progressive neuro- degenerative disease^1^. Clinical symptoms include memory impairment, aphasia, apraxia, agnosia, impairment of visuospatial skills, executive dysfunction, personality and behavioural changes, and other manifestations of dementia. Numerous complications lead to a high number of deaths. By 2025, AD patients aged 65 and over will reach 7.1 million in developed countries, an increase of nearly 29% from the 5.5 million patients of the same age in 2018. Unless there is a medical breakthrough, the number of AD patients may nearly triple from 5.5 million to 13.8 million by 2050^2^. The severity of the disease does not allow us to ignore it. An increasing number of scientists are conducting comprehensive research on the pathological mechanism of AD, but the cause has not been clearly determined.

Based on the pathological conditions of patients, various hypotheses have been proposed for the study of the aetiology of AD, including microtubule-associated protein aggregation, Aβ cascade, neuro-inflammation, and gene mutation. Some researchers believe that the central cholinergic hypothesis is the most relevant to the pathogenesis of AD. The nucleus basalis of Meynert in the basal forebrain of patients with AD is severely damaged^3^, and the content of choline is significantly increased. Severe depletion of presynaptic acetylcholine leads to severe memory loss, therefore, cholinergic agonists and cholinesterase inhibitors are consider for ameliorating cognitive deficits^4^; Others that tubulin and Aβ cascades work together leading to a series of pathological changes. Excessive Aβ in the brain of patients cannot be degraded in time, and aggregates and precipitates in the brain to form insoluble amyloids. These insoluble amyloids induce neurotoxicity^5^. It also enhances the hyperphosphorylation of tau protein, causing its abnormal aggregation intracellular neurofibrillary tangles (NFTs), resulting in the loss of normal nerve cells function and even death^6^. With further research, it has been found that homeostasis of metal ion metabolism in the body can also cause neuronal dysfunction and death^7^. Some studies have shown that excessive iron mediates the formation of Aβ plaques in the brain. And causes hyperphosphorylation of tau protein in the brain. In addition, oxidative stress is a pathogenic factor in AD. The imbalance of oxidants and antioxidants in the body leads to the increase of reactive oxygen species (ROS) and reactive nitrogen species (RNS), including H_2_O_2_ and NO^8^, which at high levels can promote Aβ aggregation and phosphorylation of tau protein^9^, and can induce mutations in neuronal DNA and RNA^10^, thereby aggravating the nerve damage. The multiple pathogenetic mechanisms are interconnected, making it impossible to accurately identify the aetiology of AD. These pathogenic mechanisms derive from pathological changes of patients with AD, and have been suggested by various studies.

The current clinical treatment drugs for AD are mainly cholinesterase inhibitors, including: donepezil, rivastigmine, galantamine; and the N-methyl-D-aspartic acid (NMDA) receptor antagonists: memantine and amantadine. These drugs relieve the symptoms of AD but lack efficacy. Researchers are now turning their attention to the development of therapeutics using natural products. Studies have shown that the structural complexity and chemical properties of natural products give them value in drug development. With the progress in scientific research, more natural product structures and properties have been confirmed, and natural products have become an important source of new lead compounds in drug research and development^11^. For example, A series of novel Lappaconitine derivatives enhanced its anti-inflammatory activity, and dihydroartemisinin derivatives and ursolic acid improved its anti-T.gondii activity^12–14^. Furthermore, natural products and their derivatives, and foodstuffs account for more than one-third of all new molecular entities approved by the FDA^15^^,^^16^. Natural products have also shown efficacy in neurodegenerative diseases. Phenylpropanoids, flavonoids, terpenoids, alkaloids, and their derivatives have been shown to improve the pathological changes of AD by acting on multiple targets, such as inhibiting acetylcholinesterase, reducing tau and amyloid aggregation, reducing neuroinflammation, reducing Aβ1-42-induced neurotoxicity, and inhibiting β-site amyloid precursor protein cleaving enzyme 1 (BACE-1). In this review, we summarise the recent five years literature on the effects of natural products on AD.

## Phenylpropanoids and their derivatives

### Ferulic acid and its derivatives

**Figure 1.**
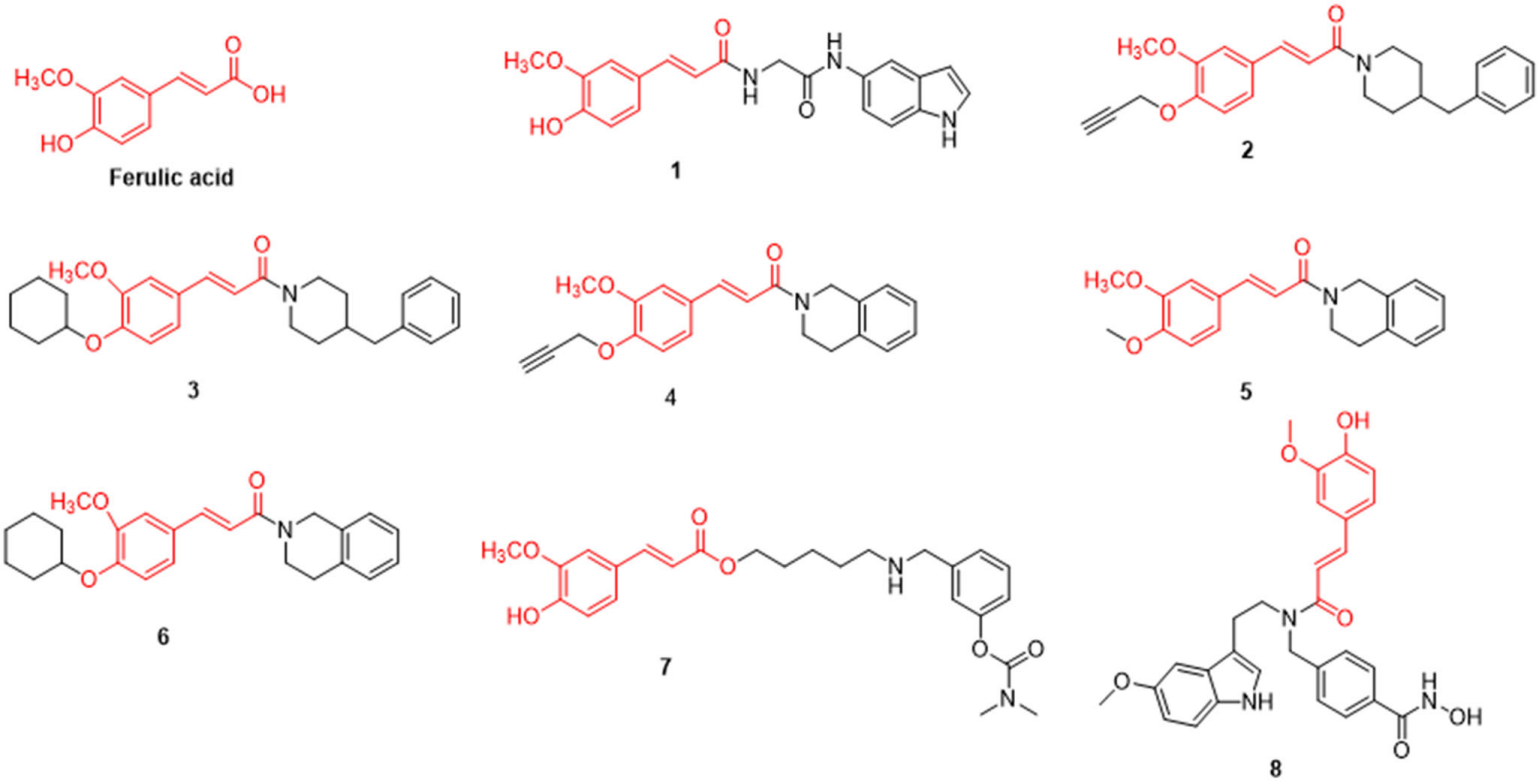
The chemical structure of ferulic acid and its derivatives **1–8**.

Ferulic acid (FA) is a phenylpropionic acid compound that has attracted the attention of many scientists for the treatment of AD. FA was found to ameliorate AD-like pathology and inhibit cognitive decline by preventing capillary hypofunction in APP/PS1 mice^17^. Treatment with the combination of two nutraceuticals, epigallocatechin gallate (EGCG) and FA, reduced brain parenchymal and cerebrovascular beta-amyloid deposition, neuroinflammation, oxidative stress, and synaptic toxicity in APP/PS1 mice^18^. *In vivo* experiments have shown that FA inhibits cholinesterase activity. Inhibition of cholinesterase can enhance cholinergic neurotransmission, thereby relieving the symptoms of AD (Figure 1).

Yash et al. synthesised several FA derivatives, and showed that compound **1** inhibited acetylcholinesterase (AChE) (IC_50_ = 5.74 μM) more strongly than the lead compound FA (IC_50_ = 15.19 μM) *in vitro* and *in vivo*. It showed good *in vivo* activity in an AD mouse model and improved spatial memory in mice with cognitive impairment in the Y-maze model. In addition, Compound **1** can effectively improve the levels of AChE and BChE in mouse models. and the introduction of an indole ring and quinoline ring improved selectivity compared to a benzene ring. They suggested that compound **1** has the potential to be developed for the treatment of AD and other neurodegenerative diseases^19^ (Figure 1, Table 1).

**Table 1.** Effect of ferulic acid derivatives on the various pathological pathways involve in AD.

Compound No.	Classification	The major targets	Activity (µM)	References
**1**	Ferulic acid derivatives	AChE inhibitory activity	IC_50_ (*h*AChE) = 5.74	^19^
**2**		MAO-B inhibitory activity Inhibits self-induced aggregation of Aβ1-42	IC_50_ (*h*MAO-B) = 0.32	^20^
**3**			IC_50_ (*h*MAO-B) = 0.56	
**4**			IC_50_ (*h*MAO-B) = 0.54	
**5**			IC_50_ (*h*MAO-B) = 0.73	
**6**			IC_50_ (*h*MAO-B) = 0.86	
**7**		Inhibits self-induced Aβ Aggregation and the efficacy of AChE	IC_50_ (*h*AChE) = 0.0192 IC_50_ (*h*BChE)= 0.66	^21^
**8**		HDAC6 inhibitory activity DPPH radical scavenging ability Cu^2+^chelating ability Preventing Aβ25 − 35 induced spatial work and long-term memory dysfunction	IC_50_ (HDAC6)=0.0307	^22^
**9**	Coumarin derivatives	MAO-B inhibitory activity	IC_50_ (*h*MAO-B) = 0.00507	^23^
**10**			IC_50_ (*h*MAO-B) =0.0042	
**11**			IC_50_ (*h*MAO-B) =0.00394	
**12**		Inhibit AChE and BChE	IC_50_ (*h*BChE) = 30.3	^24^
**13**			IC_50_ (*h*BChE) = 29.2	
**14**			IC_50_ (*h*BChE) = 37.2	
**15**			IC_50_ (*h*BChE) = 50.1	
**16**		Inhibit AChE and MAO-B Reversing cognitive dysfunction in AD mice induced by scopolamine Inhibit AChE and BChE	IC_50_ (*h*AChE) = 0.0068	^25^
**17**			IC_50_ (*h*AChE) = 0.114	
**18**			IC_50_ (*h*BChE) = 30.2	^26^
**19**			IC_50_ (*h*BChE) = 0.363	
**20**	Eugenol derivatives	MAO-A inhibitory activity	IC_50_ (*h*MAO-A) = 5.989	^27^
**21**			IC_50_ (*h*MAO-A) = 7.348	
**22**		MAO-B inhibitory activity	IC_50_ (*h*MAO-B) = 7.494	
**23**			IC_50_ (*h*MAO-B) = 9.183	

Gaofeng et al. synthesised novel *O*-alkyl ferulamide derivatives and evaluated their activity against monoamine oxidase B (MAO-B). Compounds **2**, **3**, **4**, **5**, and **6** exhibited significant selective MAO-B inhibition (IC_50_ = 0.32, 0.56, 0.54, 0.73, and 0.86 μM, respectively), significantly inhibited Aβ1-42 aggregation, and effectively protected PC12 cells Aβ1-42-induced damage, all of which have a good blood-brain barrier permeability. This is consistent with the properties required for the development of new drugs^20^ (Figure 1, Table 1).

Jin-Shuai et al. designed and synthesised 15 FA derivatives. Through *in vitro* experiments, compound **7** was found to effectively inhibit AChE and BChE (IC_50_ = 19.7 nM and 0.66 μM). respectively; when a benzylamino group with a five-carbon spacer was attached to FA, the inhibition rate of AChE was improved. Furthermore, the introduction of an electron-absorbing methoxy group to the benzene ring of FA enhanced its inhibitory activity. *h*AChE and *h*BuChE were inhibited to a greater extent when the carbamate substituent on the benzylamino group was an N-methyl group. At 20 μM, the inhibition rate of Aβ aggregation was 49.2 and PC12 cells were well protected from hydrogen peroxide-induced oxidative cell damage. Molecular docking data indicated that compound **7** is a multi-target inhibitor that interacts with both the catalytic anion site and peripheral anion site of AChE, thus exerting a dual inhibitory effect^21^ (Figure 1, Table 1).

The structure of FA and melatonin was assembled into a histone deacetylase 6 (HDAC6) inhibitor to obtain a hybrid compound **8** that can inhibit multiple targets. which can have multiple targets. It had obvious inhibitory activity against HDAC6 (IC_50_ = 30.7 nM), DPPH free radical scavenging ability similar to that of FA, and Cu^2+^ chelating ability similar to that of melatonin. Aβ25-35-induced memory impairment was reversed at lower doses. In addition, simultaneous immunomodulatory, HDAC6-selective inhibitory, and antioxidant properties were observed. Thus, it is a potential drug candidate for the treatment of neurodegenerative diseases^22^ (Figure 1, Table 1).

### Coumarin and its derivatives

**Figure 2.**
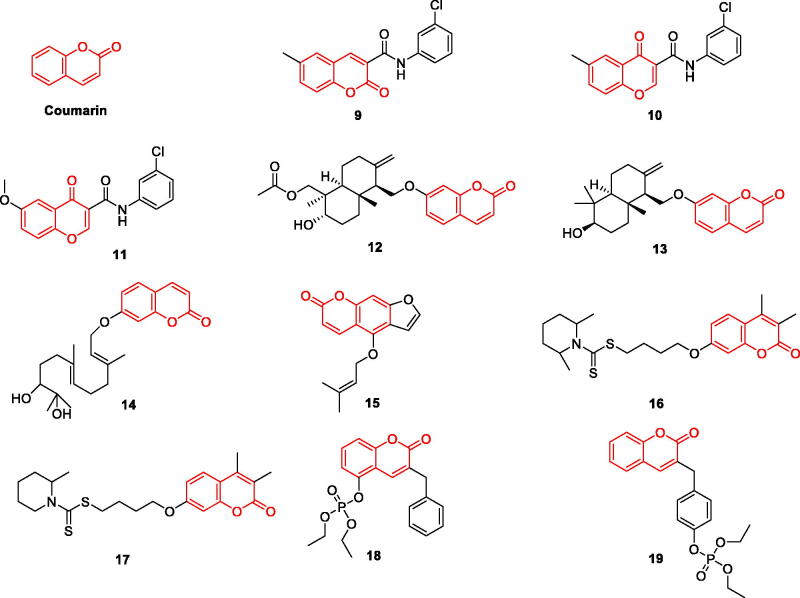
The chemical structure of coumarin and its derivatives **9–19**.

The numerous pharmacological activities of coumarins depend on their core structure (e.g. simple coumarins, fused polycyclic coumarins, and dicoumarins). At present, there have been more in-depth studies on the role of coumarin in AD. Coumarins exhibit highly diverse activities against various pathways in AD. They act as Aβ42 aggregation inhibitors, dopamine and serotonin mediators, gamma-aminobutyric acid (GABA) receptor agonists, NMDA receptor antagonists, and monoamine oxidase (MAO) inhibitors (Figure 2).

Fonseca et al. modified the structure of coumarin and chromone, and evaluated their MAO-A and MAO-B inhibitory activities. The results showed that there was no significant difference between the two natural product derivatives in their activities. They had inhibitory effect on MAO-B and no significant activity on MAO-A. The MAO-B inhibitory activity of coumarin derivative **9** (IC_50_=5.07 nM), chromone derivative **10** (IC_50_=4.2 nM) and compound** 11** (IC_50_=3.94 nM) is strong. The introduction of aniline has a good effect. The introduction of chlorine substituents at the same time can enhance the MAO-B inhibitory activity. The results of pharmacokinetics showed that compounds** 9** and **10** had good binding power and selectivity through non-competitive inhibition^23^ (Figure 2, Table 1).

Orhan et al. evaluated the activity of 17 natural coumarin derivatives on AChE and BChE. Compounds 12, 13, 14 and 15 (IC_50_ = 30.3, 29.2, 37.2, and 50.1 μM, respectively) for butyrylcholine Esterase was more inhibited than positive control galantamine (IC_50_ = 60.2 μM), structural groups and oxyanion cavities in these coumarins and peripheral anion site residues of AChE/BChE interaction, potent inhibition of BChE and AChE^24^ (Figure 2, Table 1).

He et al. studied the structure-activity relationship between coumarin and dithiocarbamate and synthesised several novel coumarin derivatives with dual targets. Compound **16** was the most effective inhibitor of AChE (IC_50_ = 68 nM). Compound **17** is a dual binding site inhibitor of AChE and a competitive inhibitor of MAO-B (0.114 µM for hAChE; 0.101 µM for hMAO-B) with a good blood-brain barrier permeability. The o-methyl substitution of piperidine on the nitrogen atom in **16** plays a key role in inhibiting the activity of AChE and MAO-B. Adding another methyl group on the piperidine ring gives compound **17**, which acquires significant MAO-B inhibitory activity. It was shown to reverse scopolamine-induced cognitive impairment in AD mice without having acute toxicity in mice at higher doses^25^. Thus, compound **17** can be used as a potential multi-target drug for the treatment of AD (Figure 2, Table 1).

Lee et al. synthesised warfarin-diethyl phosphate and coumarin-organophosphate compounds and evaluated their selectivity in inhibiting BChE *in vitro*, and concluded that the introduction of organophosphate increased ChE inhibitory activity. The selectivity of coumarin-organophosphate compounds is generally stronger than that of warfarin-diethyl phosphate for BChE inhibition; in particular,compound **19** showed a stronger inhibitory effect on BChE than **18**. A 100-fold enhanced inhibitory activity (IC_50_ = 0.363 μM) was observed^26^. It was shown that the organophosphate attached to the coumarin structure rather than the benzyl structure is more favourable for faster phosphorylation of the leaving group. The introduction of organophosphates plays a key role in enhancing the activity and increasing the binding force of the ligands (Figure 2, Table 1).

### Eugenol and its derivatives

**Figure 3.**
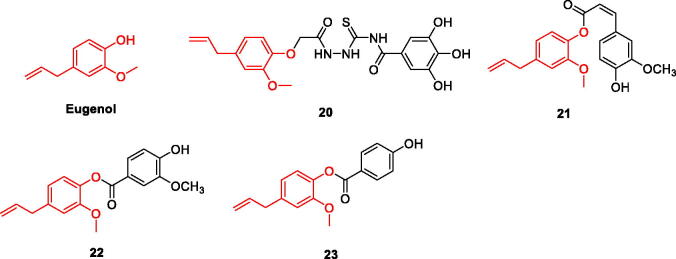
The chemical structure of eugenol and its derivatives **20–23**.

Eugenol is used to prevent and treat AD. Inhibits AChE and BChE activities in a dose-dependent manner, s showing higher antioxidant activity^28^. Studies have shown that eugenol can significantly reduce the total area of hippocampal amyloid plaques and enhance memory in male Wistar rats at both 0.01 and 0.02 mg/kg doses^29^. It also shows inhibitory activity against the MAO-B enzyme and free-radical scavenging activity^30^. The eugenol-calamus combination attenuated lipid peroxide (LPO) and AChE levels in the hippocampus and significantly improved cognitive function in streptozotocin (STZ)-induced rats^31^. Numerous studies have shown that eugenol has the potential to be developed as a multi-target directed ligand (MTDL) for the treatment of AD (Figure 3).

Based on the inhibitory effect of eugenol monomers on MAO, a series of eugenol derivatives was designed and synthesised, and their activities were evaluated. Compounds **20** and **21** were found to be the most effective hMAO-A inhibitors, with IC_50_ values of 5.989 µM and 7.348 µM, respectively, which were stronger than the positive control drug Chlorgyline (IC_50_ = 18.74 µM); selectivity index values were 0.19 and 0.14, respectively. The semicarbazide group in compound **20** was found to be important for MAO inhibition. Furthermore, the effectiveness of compound **20**, and compound **21** demonstrated the importance of the ester bond in targeting the active site of h-MAOA. Compounds **22** and **23** were more effective in inhibiting hMAO-B, with IC_50_ values of 7.494 µM and 9.183 µM, respectively, which were stronger than the control drug Pargyline (IC_50_ = 20.04 µM); selectivity indices were 5.14 and 5.72 µM, respectively; The introduction of 4-hydroxy-3- methoxybenzoate in the structure of compound **22** clearly enhanced the inhibitory activity against hMAO-B, and the smaller molecular structure of compound **23** makes it more active; therefore, it can be assumed that the introduction of the methoxy group slightly reduced the activity. The inhibition rates of the above four derivatives against the two monoamine oxidases were stronger than that of eugenol^27^. Reducing the activity of monoamine oxidase can significantly reduce oxidative stress (Figure 3, Table 1).

## Flavonoids and their derivatives

### Quercetin, kampferol and their derivatives

**Figure 4.**
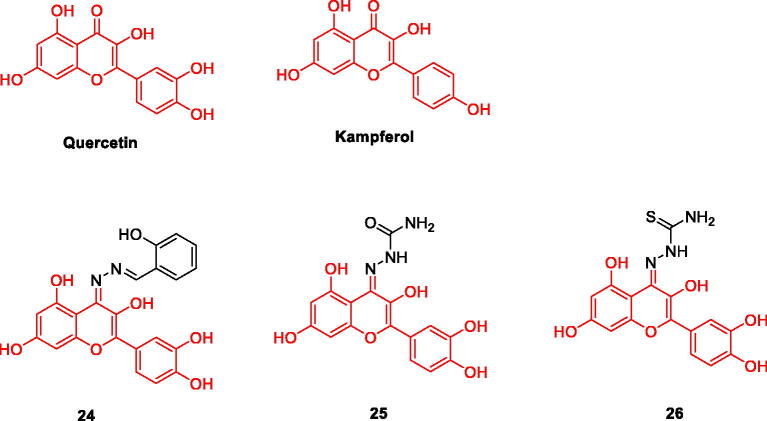
The chemical structure of quercetin /kampferol and their derivatives **24–26**.

Natural flavonoids show a large number of biological activities. At present, there is evidence that these compounds can be used as MAO inhibitors, such as Homoisoflavonoids can be used as natural scaffolds for strong and selective MAO-B inhibition^32^^,^^33^, as well as natural flavonols Quercetin and Kampferol are flavonoid alcohols that widely exist in the plant kingdom. At present, Quercetin is an expectorant drug used clinically, and also has functions such as lowering blood pressure. The similarity of their structures determines the similarity of their pharmacological effects. At present, researchers have found that Quercetin and Kampferol can be used as effective MAO-A inhibitors. Among the six compounds isolated from H.hircine, Quercetin is the only one that selectively inhibits MAO-A, with an IC_50_ value of 0.010 µM^34^. Gidaro’s research shows that Kampferol also has a strong inhibitory effect on MAO-A^35^. As effective MAO inhibitor, they can effectively protect nerve cells (Figure 4).

A series of quercetin derivatives were designed and synthesised, and their inhibitory and antioxidant activities on hMAO were screened. The results of hMAO inhibition *in vitro* showed that compound **24** was an effective hMAO-A inhibitor, while compounds **25** and **26** were effective hMAO-B inhibitors. DPPH radical scavenging activity data showed that compounds **25** and **26** showed enhanced antioxidant capacity, IC_50_ values were 5.931 µM and 6.421 µM, respectively. In addition, compounds **25 and 26** also showed significant H_2_O_2_ scavenging capacity, with IC_50_ values of 5.80 µM and 6.20 µM, respectively. In the docking study, compounds **25** and **26** have good correlation with experimental MAO. This explains the specific inhibition of MAO activity and antioxidant activity. These compounds can be used as effective neuroprotective agents and antioxidants to treat AD, so further experimental research is particularly important for clinical application^36^ (Figure 4, Table 2).

**Table 2.** Effect of flavonoids derivatives on the various pathological pathways involve in AD.

Compound NO.	Classification	The major targets	Activity (µM)	References
**24**	Quercetin derivatives	MAO-A inhibitory activity MAO-B inhibitory activity DPPH radical scavenging activity The hydrogen peroxide scavenging activity	IC_50_ (*h*MAO-A) = 13.1 IC_50_ (*h*MAO-B) = 46.11	^36^
**25**			IC_50_ (*h*MAO-A) = 32.16 IC_50_ (*h*MAO-B) = 18.87	
**26**			IC_50_ (*h*MAO-A) = 36.92 IC_50_ (*h*MAO-B) = 12.70	
**27**	Isoliquiritigenin derivatives	5-LO inhibitory activity Inhibits self induced aggregation of Aβ1-42	IC_50_ (Aβ1-42) = 3.2	^37^
**28**			IC_50_ (Aβ1-42) = 2.2	
**29**			IC_50_ (Aβ1-42) = 5.9	
**30**			IC_50_ (Aβ1-42) = 14.6	
**31**	Liquiritigenin derivatives	Selectively inhibit AChE and BChE The radical scavenging effects	IC_50_ (*h*AChE) = 0.03 IC_50_ (*h*BChE) = 5.46	^38^
**32**	Chalcone derivatives	Inhibit *h*MAO-A and *h*MAO-B	IC_50_ (*h*MAO-B) = 0.0044	^39^
**33**			IC_50_ (*h*MAO-A) = 4.9 IC_50_ (*h*MAO-B) = 0.0051	
**34**		Inhibit AChE and BChE Metal chelating agent Inhibit Aβ1-42 aggregation Improve memory impairment induced by scopolamine	IC_50_ (*h*AChE) = 1.3 IC_50_ (*h*BChE) = 1.2	^40^
**35**		AChE inhibitory activity BACE-1 inhibitory activity	IC_50_ (*h*AChE) = 0.08 IC_50_ (BACE-1) = 2.71	^41^

### Liquiritigenin and isoliquiritigenin and their derivatives

**Figure 5.**
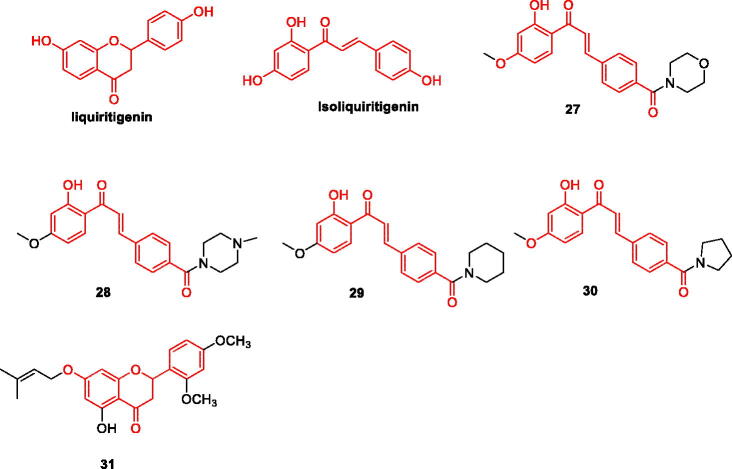
The chemical structure of liquiritigenin/isoliquiritigenin and its derivatives **27–31**.

Both liquiritigenin and isoliquiritigenin are dihydro- flavonoid monomer compounds extracted from the natural plant liquorice. As these two compounds have similar structures, their pharmacological activities are also similar. In recent years, researchers have found that liquiritigenin and isoliquiritigenin are promising agents for the treatment of AD. Studies have shown that liquiritigenin can be a potent inhibitor of tau amyloid fibril formation by preventing structural transitions in its structure and exposure of hydrophobic groups. Therefore, reducing tau aggregation-mediated neurotoxicity^42^. Aβ levels can also be reduced by modulating the M1/M2 phenotype transition in microglia, thereby reducing memory decline during AD^43^. Isoliquiritigenin attenuated Aβ25-35 induced neuronal damage in rat cortical neurons by interfering with [Ca^2+^]i and ROS production^44^. Isoliquiritigenin reduces neuronal damage by inhibiting 5-lipoxygenase (5-LO). The activity of 5-LO is regulated by 5-lipoxygenase-activating protein (FLAP), and targeting the 5-LO/FLAP pathway is considered an effective strategy for the treatment of AD. 5-LO is involved in AD pathological changes, and its activity is significantly enhanced. Studies have shown that activation of 5-LO promotes Aβ amyloid deposition and tau hyperphosphorylation^45^ (Figure 5, Table 2).

Yi-Ping et al. designed and synthesised a series of isoliquiritigenin derivatives, most of which were more potent than the positive control drugs (resveratrol and nordihydroguaiaretic acid) on Aβ1-42 aggregation and 5-LO. More potent and similar to the lead compound isoliquiritigenin. In addition, it is interesting that compounds **27–30** have a strong inhibitory effect on the above two targets, and the structure-activity relationship shows that substitution at the 4-position of the A ring can improve the inhibitory activity of the compound, especially the six-membered ring amino side chain substituent. Among them, compound **27** (Figure 5) has the strongest inhibitory effect on the two targets, and the IC_50_ value of Aβ1-42 aggregation inhibition was 3.2 µM, which was stronger than that of the positive control drugs resveratrol (IC_50_ = 15.9 µM) and isoliquiritigenin (IC_50_=19.7 µM); the inhibition IC_50_ of 5-LO was 6.1 µM, which was stronger than that of the positive control drugs Noidihydroguaisretic acid and isoliquiritigenin, with IC_50_ of 12.4 µM and 18.6 µM, respectively. Molecular docking results showed that van der Waals forces and hydrogen bonding play important roles in the stability of **27**/Aβ1-42 complexes and the intermolecular hydrogens bonding interaction between compound **27** and 5-LO. Therefore, compound **27** can be further studied as a potential drug for the treatment of AD^37^ (Figure 5, Table 2).

Selectivity of AChE and BuChE Based on Liquiritigenin, 7-prenyloxy-2,3-dihydroflavanone derivatives and 5-hydroxy-7- prenyloxy-2,3-dihydroflavanone derivatives, among 32 synthesised derivatives, compound **31** .It was confirmed to inhibit AChE (IC_50_ = 0.09 µM) and BChE (IC_50_ value of 5.46 µM) significantly stronger than donepezil (IC_50_ = 0.48 and 10.60 µM), through dual-site binding ability^38^. The structure-activity relationship of the two derivatives was discussed. The structure-activity relationships of the two derivatives are discussed. The introduction of the 5-position hydroxyl group may enhance the hydrophilicity of the molecule, and the introduction of the methoxy group improved inhibitory activity. Dihydroflavanones can bind to peripheral anion sites, causing some molecules to tilt and bind more tightly with the catalytic active site of acetylcholinesterase, effectively inhibiting its activity (Figure 5, Table 2).

### Chalcone and its derivatives

**Figure 6.**
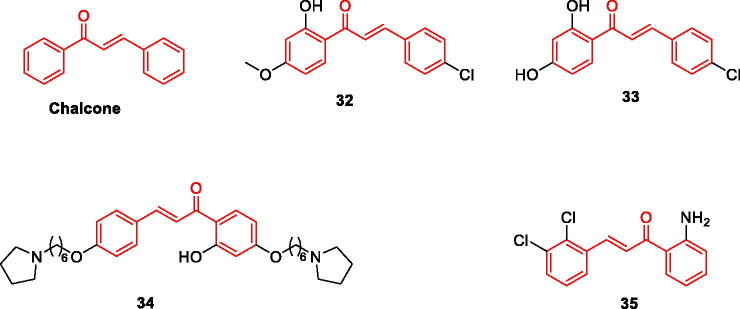
The chemical structure of chalcone and its derivatives **32–35**.

Chalcone, also known as diphenylpropenone, is a flavonoid composed of phenolic compounds, one of the largest groups of bioactive natural products. Its unique chemical structural features have inspired the synthesis of numerous chalcone derivatives. To explore their ability to reduce intracellular amyloid and associated oxidative damage, more than ten natural products, including chalcone, were assayed, and trans-chalcone and baicalein were found to be the most potent compounds^46^. All compounds were flavonoids. Six natural chalcones were assayed, and most compounds exhibited inhibitory effects against both MAO-B and AChE^47^. Chalcone has a lower molecular weight, structural modification on its skeleton can increase the interaction with the target. Chalcone is more suitable for multi-target drug molecular design. This can greatly improve biological activity (Figure 6).

Based on the inhibitory activity of monoamine oxidase, Chimenti et al. designed and synthesised a series of chalcone derivatives, all of which showed inhibitory activity. Compound **32** (IC_50_=4.4 nmol) showed the strongest inhibitory effect on MAO-B; Compound **33** showed inhibitory activity on MAO-A and MAO-B, IC_50_ was respectively 4.9 μM and 0.0051 μM. The results of molecular docking confirmed the experimental results *in vitro*. Compound **32** interacts effectively with tyrosine amino acid residues, and compound **33** shows interaction through its chlorine phenyl ring and two hydrogen bonds with other related residues. This also explains the source of the inhibitory effect of the two compounds. It is also confirmed that chalcone can be used as an important source of hMAOs inhibitors skeleton^39^ (Figure 6, Table 2).

Ping et al. designed and synthesised a series of novel chalcone-*O*-alkylamine derivatives with anti- AD activity based on the concept of multi-target therapeutic drugs. The experimental results showed that the compounds have multi-target therapeutic effects: (1) Inhibit both AChE and BChE; (2)Inhibit the expression of MAO-B; (3)Inhibit self-induced Aβ_1-42_ aggregation; (4) Be used as selective metal chelators. Among them, the comprehensive experimental results found that compound **34** had the highest potential. The IC_50_ values for the inhibition of AChE and BuChE were 1.3 μM and 1.2 μM, respectively, and inhibitory activity on the expression of MAO-B was the strongest (IC_50_ = 0.57 μM). It exhibits significant inhibition and depolymerisation of Aβ1-42 aggregation and can also inhibit and break Cu^2+^-induced Aβ1-42 aggregation. Structure-activity relationship analysis showed that the length of the methylene chain has a key effect on the inhibition of AChE and BuChE, and elongation of the carbon chain significantly increases the inhibitory effect^40^ (Figure 6, Table 2).

Renata et al. synthesised a series of 2′-aminochalcone derivatives for the inhibition of AChE and BACE-1 enzymes and Aβ. All amino-modified chalcones were found to have a better inhibitory effect on BACE-1 enzyme, which can reduce the production and deposition of amyloid. Furthermore; compound **35** had a strong inhibitory effect on multiple targets; the IC_50_ value for ACHE was 0.08 μM, and the IC_50_ value for BACE-1 was 2.71 μM. The molecular docking results verified the *in vitro* experiments, and the compound can be used as a dual-target drug candidate for AD^41^ (Figure 6, Table 2).

## Terpenoids and their derivatives

### Triptolide and its derivatives

**Figure 7.**
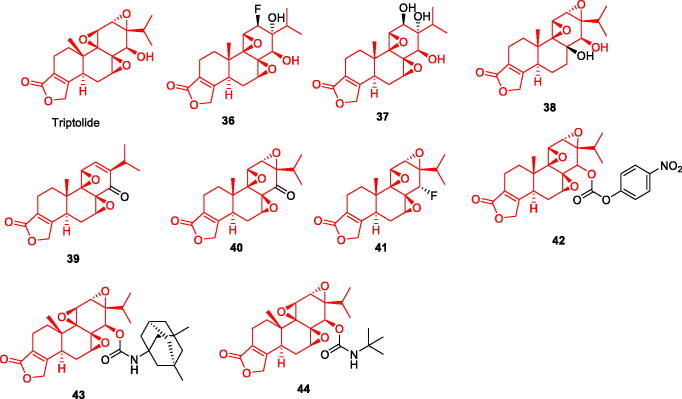
The chemical structure of triptolide and its derivatives **36–44**.

Triptolide is an epoxy diterpene lactone and one of the main active components of Tripterygium wilfordii. Triptolide can inhibit the activation and proliferation of microglia and astrocytes in an APP/PS1 double-transgenic AD mouse model reduce neurotoxicity^48^. Furthermore, it was found to inhibit the expression of β-site amyloid precursor protein cleaving enzyme 1 and reduce Aβ production and deposition in the brain thus reversing the pathological changes in AD^49^. *In vivo* experiments have shown that triptolide increases the expression of insulin-degrading enzyme, a major Aβ-degrading enzyme in the brain^50^, which can reduce AD pathological symptoms by increasing Aβ degradation. A number of studies have confirmed that the natural product triptolide is an effective and multifunctional lead compound that can act on multiple targets relevant to AD and has the potential to be developed for the treatment of AD (Figure 7).

Some researchers have synthesised 9 triptolide derivatives based on structure-activity relationship analysis. Cell culture experiments were carried out, and it was concluded that compound **43** had the greatest potential for further study. Compounds **36–38** are modified by ring-opening C12 and C13-epoxy structure of triptolide; compound **39** is modified by ring-opening C-7 and C8-β-epoxy structure; compounds **40–44** are modified by ring-opening of 14 -β-OH for modification. *In vitro* experiments showed that triptolide could protect cells from Aβ1-42 damage, and the ring-opening of its epoxy group enhanced its cell damage. indicating that the neuroprotective pharmacophore of triptolide is epoxy group rather than 14-β-OH. Compound **43** dose-dependently enhanced the survival rate of Aβ1-42-treated cells. It also had the strongest anti-inflammatory activity, similar to triptolide, and completely inhibited LPS-induced TNF-α expression at a concentration of 10 nM, which warrants further study^51^ (Figure 7, Table 3).

**Table 3.** Effect of terpenoids derivatives on the various pathological pathways involve in AD.

Compound No.	Classification	The major targets	Activity (µM)	References
**36–44**	Triptolide derivatives	Protect nerve cells from Aβ1-42 damage Inhibits LPS induce TNF	_	^51^
**45/46**	Andrographolide derivatives	Inhibits LPS induced NO production, iNOS expression and proinflammatory cytokine TNF- α and IL-6 Protect neurons from microglia mediated neurotoxicity Promotes NGF induced axon growth in PC12 cells	_	^52^
**47-49**		β-Secretase inhibitors AChE inhibitory activity	_	^53^
**50**	Glycyrrhetinic acid derivatives	AChE inhibitory activity	IC_50_ (*h*AChE) = 3.43	^54^
**51**			IC_50_ (*h*AChE) = 5.39	
**52**			IC_50_ (*h*AChE) = 6.27	
**53**			IC_50_ (*h*AChE) = 8.68	
**54**		BChE inhibitory activity	IC_50_ (*h*BChE) = 5.43	^55^
**55**			IC_50_ (*h*BChE) = 9.81	
**56**	Safranal derivatives	MAO-A inhibitory activity MAO-B inhibitory activity	IC_50_ (*h*MAO-A) = 9.93 IC_50_ (*h*MAO-B) = 0.0091	^56^

### Andrographolide and its derivatives

**Figure 8.**
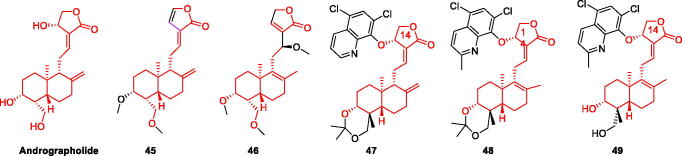
The chemical structure of andrographolide and its derivatives **45–49**.

Andrographolide (ANDRO) is a diterpene lactone compound extracted from Andrographis paniculata, which is one of the main components of the traditional Chinese medicine Andrographis paniculata, and is known as a natural antibiotic drug. Therefore, it is widely used in clinical practice^57^. However, many studies have shown that ANDRO can be a drug for AD through multiple pathways. For example, ANDRO activated autophagy-related genes and proteins (Beclin-1 and LC3), while it also enhanced the expression of Nrf2 and p62 at the mRNA and protein levels, and decreased p-tau and p-tau in Aβ1-42 stimulated cells. Thus improving cell damage caused by Aβ1-42 injury^58^; ANDRO has also been shown to stimulate neurogenesis in the hippocampus and improve memory performance by inducing the proliferation of neural precursor cells; ANDRO induces glucosamine-nitrosourea compound behavioural disorders and changes in hippocampal biochemistry, neuroinflammatory mediators, and neurotransmitters have protective effects^59^. In addition, ANDRO reduced the levels of Aβ1-42 and p-tau in the rat hippocampus, thereby exerting neuroprotective effects and ameliorating cognitive impairment and AD-like symptoms^60^. ANDRO can improve the pathological symptoms of AD in various ways, and more in-depth and comprehensive research may bring ANDRO to the clinic (Figure 8).

Xu et al. designed and synthesised 12 Andrographis paniculata derivatives, all of which were non-toxic. Screening for inhibitors of LPS-induced NO production in BV-2 cells showed that compounds **45** and **46** had stronger inhibitory rates than the lead compound ANDRO, suggesting that the introduction of C-3 and C-19 acetyl groups has a major effect. compounds **45** and **46** on LPS-induced proinflammatory factors TNF-α and IL-6 significantly attenuated the levels of LPS-induced pro-inflammatory factors TNF-α and IL-6 in a dose-dependent manner. These results suggest that compounds **45** and **46** can reduce the production of pro-inflammatory factors and protect neuronal cells from microglia-mediated neurotoxicity^52^ (Figure 8, Table 3).

Dey et al. synthesised 24 andrographolide derivatives, which can be divided into two series according to their conformation at the C-14 position. BACE-1 cleaves β-amyloid precursor protein (βAPP) to produce Aβ, while α-secretase prevents Aβ production and triggers the release of neuroprotective sAPPα^61^. ANDRO and compound **47** were found to activate α-secretase, and compounds **48** and **49** to effectively inhibit BACE-1, thus all inhibiting the production of Aβ^53^. Therefore, other cascade reactions caused by Aβ are reduced and damage to nerve cells is prevented (Figure 8, Table 3).

### Glycyrrhetinic acid and its derivatives

**Figure 9.**
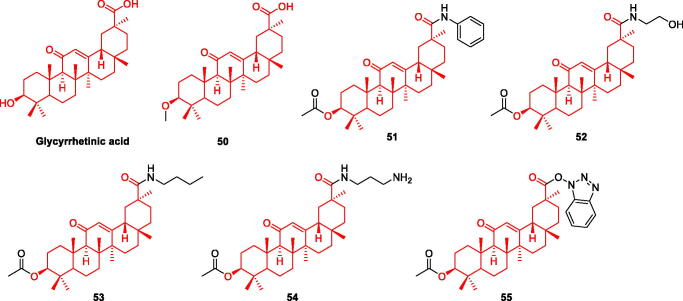
The chemical structure of glycyrrhetinic acid and its derivatives **50–55**.

Glycyrrhetinic acid also has potential for the treatment of AD. Both 18α- and 18β-glycyrrhetinic acids exert different degrees of inhibition of BACE1. 18β-Glycyrrhetinic acid showed a strong inhibitory effect with IC_50_ of 8.93 µM^62^. Furthermore, it decreased the expression levels of BACE-1 and reduced Aβ production Studies have shown that the coumarins glycerol and liquiritigenin, isolated from liquorice, have cholinesterase inhibitory effects^63^. Some triterpenoids, such as oleanolic acid and ursolic acid, have been shown to inhibit acetylcholinesterase^64^, Based on this, many researchers have modified the structure of glycyrrhetinic acid to produce acetylcholine inhibitors with higher inhibitory activities (Figure 9).

Fatma et al. synthesised a series of glycyrrhizic acid derivatives, and evaluated the AChE inhibitory effect of 13 derivatives and glycyrrhizin using the Ellman method. They found that **50, 51, 52** and **53** had higher inhibitory effects than glycyrrhizin (IC_50_ = 3.43, 5.39, 6.27 and 8.68 μM, respectively). From these data, The inhibition rate of C-30 forming amide bond is stronger than that of forming ester bond. No cytotoxicity was observed in the normal cells (WI-38). Pharmacological experiments indicated that the compounds had insignificant antioxidant capacity; thus, their effect is not through the antioxidant pathway^54^. The complete mechanism of action is not yet clear, and further in-depth research is required (Figure 9, Table 3).

Stefan et al. synthesised a series of aminoglycyrrhetinic acid derivatives and discussed the structure-activity relationship at C-3 and C-30 positions. The first group of derivatives is to synthesise 27 amino acids-glycyrrhetinic acid by esterification of *N*-Boc-amino acids with C-3 position. The second group is glycyrrhetinic acid and the corresponding brominated and alkyl diamines to form ester and amide derivatives; the third group is the reaction of glycyrrhetinic acid C-30 and nitrogen-containing heterocycles with larger groups, and two derivatives were synthesised. *In vitro* experiments showed that the inhibitory effect of the first group of derivatives on AChE was stronger than that of BChE. Compounds **54** and **55** had strong selective inhibition on BChE, with IC_50_ of 5.43 and 9.81 μM, respectively. There was an interaction between the two compounds and the active site of BChE^55^. The results of molecular docking confirmed the results of cell experiments (Figure 9, Table 3).

### Safranal and its derivatives

**Figure 10.**
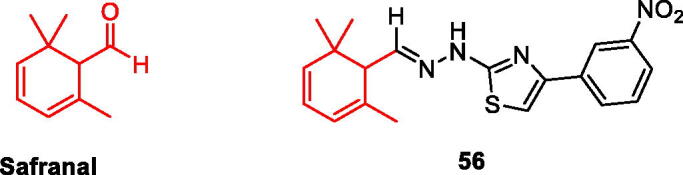
The chemical structure of safranal and its derivatives **56**.

Saffron is a perennial flower of crocus in Iridaceae, also known as saffron, which is most commonly used in spices. Saffron and its main components can improve mental diseases and neurodegenerative diseases, among which Safranal is the main component of saffron, a monoterpenoid aldehyde. At present, Safranal can reverse the neurotoxicity of PC12 cells induced by OGD by regulating oxidation and apoptosis^65^; Reduced Aβ and H_2_O_2_ induced PC12 cytotoxicity and oxidative damage^66^; Interacted with cholinergic, glutamic and dopaminergic systems^67^ (Figure 10).

Carradori et al. evaluated the hMAO activity of a series of saffron aldehyde derivatives, and found that safranal had no inhibitory effect on hMAO, but its derivatives showed good inhibitory effect, especially **compound 56** on hMAO-A (IC_50_ = 9.93 μM) And hMAO-B (IC_50_ = 0.091 μM) All showed inhibition, especially for MAO-B, its inhibition effect could be compared with lazabemide (IC_50_ = 0.01 μM). It’s on a par. **Compound 56** can be used as a new skeleton of hMAO inhibitors. It can be seen that the structure of Safranal may be transformed into a highly effective hMAO-B inhibitor and a moderately effective hMAO-A inhibitor^56^ (Figure 10, Table 3).

## Saponin and its derivatives

### Diosgenin and its derivatives

**Figure 11.**
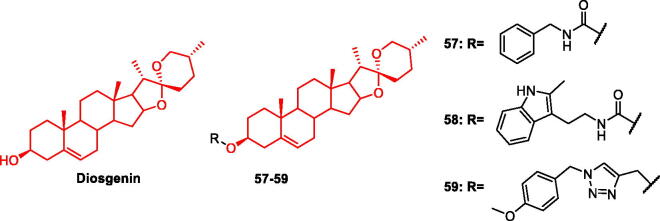
The chemical structure of diosgenin and its derivatives **57–59**.

Diosgenin is an important natural steroidal sapogenin derived from a wide range of sources, including fenugreek. It is an important raw material for the synthesis of steroid hormone drugs such as cortisone. Diosgenin protected a rat model of AD from Aβ1-42-induced neurotoxicity^68^. Diosgenin was found to reduce heat shock homologs in Aβ-induced injury in 5XFAD mice^69^, HSC70 belongs to the family of heat shock proteins and can promote neuronal axonal degeneration and pathology in the brain of AD patients when expressed at high levels^70^, Τhus, effectively inhibiting HSC70 as a new target for the treatment of AD (Figure 11).

Based on the fact that diosgenin can interact with multiple targets, many scientists have modified its structure, hoping to obtain anti-AD drug candidates with stronger activity and fewer side effects. Gui-Xiang et al. added carbamates to the diosgenin structure and examined the structure-activity relationship. Studies have shown that carbamate fragments can significantly improve AD pathology. Therefore, this topic has become a research hotspot. Numerous studies have reported on the treatment of AD^71^. Antioxidative, anti-inflammatory, and anti-Aβ1-42 activities of the obtained diosgenin carbamate derivatives were evaluated. Compound **57** protected nerve cells from H_2_O_2_-induced damage. A ten-fold stronger protective effect than the lead compound diosgenin was observed; it significantly reduced the production of pro-inflammatory factors such as TNF-α, IL-6, IL-1β, and NO, and reduced Aβ1-42-induced nerve cell damage in a dose-dependent manner^72^ (Figure 11, Table 4).

**Table 4.** Effect of saponin derivatives on the various pathological pathways involve in AD.

Compound NO.	Classification	The major targets	Activity	References
**57**	Diosgenin derivatives	Protect the nerve cells of ageing mice with subcutaneous injection of D-galactose Reduced IL-1β, IL-6 and TNF-a levels	_	^72^
**58**		Reduced ROS production induced by H_2_O_2_ Reduces 6-OHDA, H_2_O_2_, Aβ1-42 induced cell damage	Neuroprotective effects against H_2_O_2_: 52.9% Neuroprotective effects against 6-OHDA: 38.4% Neuroprotection activity against Aβ1-42 damage: 54.4%	^73^
**59**		Reduces neurotoxicity induced by oxygen glucose deprivation and NO production induced by LPS	NO inhibition %(10 μM): 40.4%	^74^
**60**	Sarsasapogenin derivatives	Improved learning and memory impairment of mice injected with Aβ1-42 Protect PC12 cells from Aβ1-42induced damage	The viability of H2O2-treated PC12 cells (10 µM): 36.6%	^75^
**61**			The viability of H2O2-treated PC12 cells (10 µM): 43.9%	
**62**		Inhibit Aβ1-42 aggregation Reduce H_2_O_2_ induced SH-SY5Y cell neurotoxicity	Inhibition of Aβ1-42 aggregation: 84.74%	^76^
**63**			Inhibition of Aβ1-42 aggregation: 75.06%	

Given that indole skeleton has been widely considered to have neuroprotective effects, Li-Cheng et al. synthesised 19 diosgenin-indole derivatives. Some compounds were modified by the introduction of carbamate fragments, while others used ester bonds as linkers, and the data showed that carbamate fragments attributed more neuroprotective properties than the ester bonds. All compounds were tested for neuroprotective effects against H_2_O_2_ (hydrogen peroxide), 6-OHDA (6-hydroxydopamine), and Aβ1-42. It is known that compound **58** can protect against nerve damage caused by the three agents, and compound **58** can reverse the cell damage of H_2_O_2_ (52.9%), 6-OHDA (38.4%), and Aβ1-42 (54.4%). was the most potent compound. Molecular docking also showed that **58** had good affinity for Aβ1-42 and could interact with it. In the water maze experiment, Compound **58** significantly improved cognition and memory in Aβ1-42-injured mice. Therefore, Compound **58** can be considered a potential multi-target drug for AD^73^ (Figure 11, Table 4).

The introduction of triazole fragments significantly enhanced the neuroprotective activity of diosgenin. Diosgenin-phenyl/benzyltriazole derivatives were designed and synthesised, and their structure-activity relationship was examined. The activity of benzyl triazole was stronger than that of phenyl triazole. Compound **59** showed the strongest inhibitory effect on NO production at 10 μM, reducing neuroinflammation. Neuroprotective activity reached 73.9 μM, which was stronger than diosgenin (3.5 μM). Compound **59** has the potential for further research in the fight against AD^74^ (Figure 11, Table 4).

### Sarsasapogenin and its derivatives

**Figure 12.**
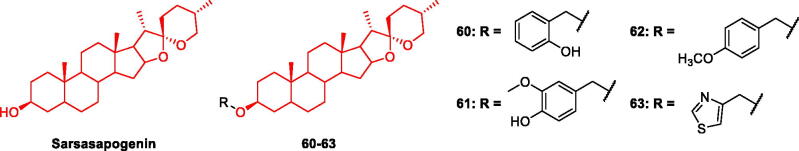
The chemical structure of sarsasapogenin and its derivatives **60–63**.

Sarsasapogenin is a steroidal sapogenin isolated from the Chinese herbal medicine Anemarrhena. It is similar in structure to diosgenin and has a similar structure-activity relationship. Current studies have shown that sarsasapogenin significantly inhibits key enzymes involved in AD pathogenesis, namely AChE, BuChE, BACE1, and MAO-B^77^^,^^78^, in a concentration-dependent manner. It also inhibits neuroinflammation and Aβ amyloid production^79^^,^^80^. It can exert neuroprotective effects in many ways, thereby improving pathological symptoms. Derivative design has also received considerable attention. Based on the activity of lead compounds, the synthesis of potential AD drugs with stronger activity and minimal side effects has also been extensively studied (Figure 12).

The introduction of *N*-substituted piperazine carboxylic acid groups significantly enhanced anti-inflammatory and antioxidant activities. Sarsasapogenin can be considered a potential building block for AD drug design^81^. Gui-Xian et al. evaluated sarsasapogenin-*N*-substituted piperazine carboxylic acid derivatives and found that compounds **60** and **61** significantly increased the viability of H_2_O_2_-treated PC12 cells by 36.6 and 43.9%, respectively; compounds **60** and **61** of 10 μM showed high NO inhibition rates of 15.2 and 24.41%, respectively^75^ (Figure 12, Table 4).

Wenbao designed and synthesised a novel sarsasapogenin-triazole hybrid and examined its structure-activity relationship. The introduction of 1,4-substituted triazolyl groups significantly enhanced their activity. Compounds **62** and **63** had a good inhibitory effect on Aβ aggregation, with inhibition rates of 84.74 and 75.06%, respectively, which were higher than those of the positive control drug curcumin (55.87%), and reduced Aβ-induced neuronal damage. In addition, the learning and memory abilities of model mice improved^76^. Therefore, the sarsasapogenin skeleton can serve as a basic structure for the development of anti-AD drugs (Figure 12, Table 4).

## Alkaloids and their derivatives

### Piperine and its derivatives

**Figure 13.**
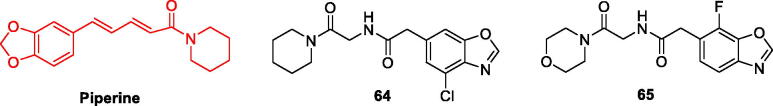
The chemical structure of piperine and its derivatives **64–65**.

Piperine is a piperidine alkaloid with a relatively simple structure and one of the main physiologically active components of piperine and longan. Piperine is currently used clinically as a spectral anticonvulsant. In addition, Masoomeh et al. showed that long-term treatment with piperine reduced oxidative damage of rats intracerebroventricularly (ICV) injected with STZ^82^. Furthermore, it reduced the synaptic toxicity of STZ in the hippocampus, thereby exerting a neuroprotective effect. Suresh et al. showed that intraperitoneal injection of piperine in diabetic rats not only improved memory in diabetic rats, but also reduce AD-related BACE1, PSEN1, APAF1, CASPASE3, and CATALASE gene expression^83^. Hsieh et al. also found that piperine protects hippocampal neurons by upregulating protein kinase B (Akt) and glycogen synthase kinase 3β (GSK-3β) signalling pathways in the hippocampus and reducing kainate (KA)- induced excitotoxicity^84^. Studies have also shown that piperine can inhibit the pathological changes in AD in multiple ways and can be used as a potential anti-AD drug (Figure 13).

Xiping et al. synthesised Compound **64**. Through *in vitro* experiments, it was shown that Compound **64** binds strongly to Keap-1 and activate the Keap1-Nrf2-ARE signalling pathway *in vitro*^85^. This signalling pathway is important targets of AD^86^^,^^87^. The activation of Nrf2/ARE and inhibition of Keap1 can play a strong role in the treatment of AD. The activation of Nrf2 increases the expression levels of superoxide dismutase, catalase, and glutathione peroxidase, thereby reducing oxidative stress^88^. Inhibition of Keap1 increases Nrf2 expression thus reducing oxidative stress, which plays a key role in AD pathology. Ibotenic acid (IBO), an NMDA receptor agonist, induces excitatory cholinergic dysfunction^89^. Compound **64** effectively prevented IBO-induced cognitive impairment, elevated acetylcholine levels, increased neurotransmission, and reversed cholinergic neuronal damage^85^ (Figure 13, Table 5).

**Table 5.** Effect of alkaloid derivatives on the various pathological pathways involve in AD.

Compound NO.	Classification	The major targets	Activity	References
**64**	Piperine derivatives	Inhibiting apoptotic cell death induced by IBO Inhibited the interaction between Keap1 and Nrf2	_	^85^
**65**		Inhibits the increase of Bax/Bcl2 ratio protects hippocampal neurons Inhibits the activation of NLRP3 inflammatory bodies in hippocampus Reduces Aβ1-42 Induced neurotoxicity in SH-SY5Y cells	_	^90^
**66**	Harmine derivatives	Inhibits GSK-3β and DYRK1A, Inhibits oka induced tau hyperphosphorylation in SH-SY5Y cells,	IC_50_ (GSK-3β) = 71 nM IC_50_ (DYRK1A) = 103 nM	^91^
**67**		AChE inhibitory activity BChE inhibitory activity Inhibits GSK-3β	IC_50_ (*h*AChE) = 0.27 µM IC_50_ (*h*BChE) = 20.82 µM IC_50_ (GSK-3β) = 6.78 µM	^92^
**68**	Oxymatrine derivatives	Protect PC12 cells from Aβ induced damage Inhibition of LPS induced NO	_	^93^
**69**				
**70**				

The research group also synthesised a piperine derivative compound **65**. *In vivo* and *in vitro* experiments showed that compound **65** (1) Inhibited the formation of the Keap1-Nrf2 complex and reduced oxidative stress; (2) Reduced neurotoxicity induced by Aβ1–42 in SH-SY5Y cells and improved Aβ-induced cognitive impairment; and (3) Reduced expression levels of NLRP3, IL-1β and TNF-α inflammatory factors and resolved neuroinflammation. Therefore, compound **65** can be used to treat AD through various pathways, such as reducing oxidative stress, neurotoxicity, and neuroinflammation^90^ (Figure 13, Table 5).

### Harmine and its derivatives

**Figure 14.**
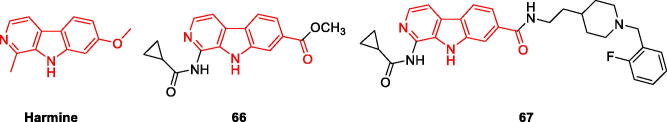
The chemical structure of harmine and its derivatives **66–67**.

Harmine alkaloids (HARs) are β-carboline alkaloids extracted from HAR officinalis seeds, and their molecular skeleton contains a pyridoindole structure. HARs have a variety of pharmacological activities, including antitumor, anti-inflammatory, antiviral, and anti-AD effects. Research has shown that HARs can act on multiple targets to improve the symptoms of AD. *In vivo* experiments, HARs were found to selectively inhibit AChE and MAO-A^94^. Dandan et al. showed that HAR has good permeability across the blood-brain barrier. *In vitro* experiments have shown that it can inhibit AChE activity. Molecular docking data also showed that it is tightly bound to the catalytic site of AChE^95^. A study by Yunpeng et al. showed that the combination of HAR and memantine (MEM) increases efficacy in the treatment of AD. The results from the pharmacokinetic experiments showed that the elimination rate of HARs, MEM, and HOL (the main metabolite of HARs) was slowed down after combination treatment, improving bioavailability and achieving therapeutic concentrations^96^ (Figure 14).

Wenwu et al. obtained two series of compounds with significantly enhanced neuroprotective effects by the structural modification of HAR. Among these, compounds **66** and **67** exhibited the best activity. Pharmacological experiments showed that the compound **66** exhibited strong inhibitory effects on GSK-3β and dual-specificity tyrosine phosphorylation-regulated kinase 1 A (DYRK1A), with IC_50_ values of 71 and 103 nM, respectively^91^. GSK-3β is involved in tau hyperphosphorylation^97^, and DYRK1A is an upstream kinase of GSK-3β signalling. First, DYRK1A is phosphorylated and GSK-3β phosphorylates tau protein. Therefore, inhibiting the expression of GSK-3β and DYRK1A can reduce tau hyperphosphorylation, effectively preventing the formation of NFTs^98^, and reducing nerve damage. Experiments have shown that compound **66** is a dual-target inhibitor, which greatly reduces the hyperphosphorylation of tau protein and prevents nerve cell damage. It can be used as a dual-target drug candidate for the effective treatment of AD (Figure 14, Table 5).

The second series was developed by introducing the *N*-benzylpiperidine group. All compounds were found to have significant anti-AChE activity and good inhibitory activity against BChE. Among them, compound **67** had the best effect, with IC_50_ of 0.27 and 20.82 μM for AChE and BChE, respectively. It also inhibited GSK-3β with an IC_50_ of 6.78 μM. From these data, it can be concluded that the linkage of two carbon atoms between the 7-position β-carboline and *N*-benzylpiperidine is more suitable for increasing the inhibitory activity towards AChE. Molecular docking data showed that compound **67** was tightly bound to both the catalytic active site and peripheral anionic site of AChE, and also formed a stable interaction with GSK-3β. It has been demonstrated that **67** can exert anti-AD effects by inhibiting AChE, BChE, and GSK-3β^92^ (Figure 14, Table 5).

### Oxymatrine and its derivatives

**Figure 15.**
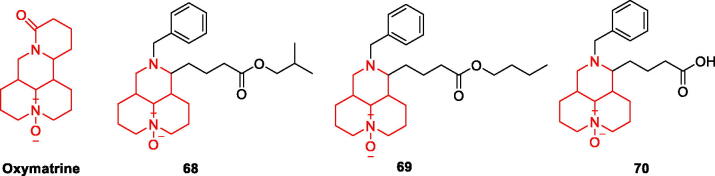
The chemical structure of oxymatrine and its derivatives **68–70**.

Oxymatrine (OMT) is a quinazoline alkaloid extracted from the root of *Sophora flavescens*, which proves that OMT can reduce Aβ1–42-induced neuronal damage and inhibit the activation of microglia by inhibiting NF-κB and MAPK signalling pathways and anti-neuroinflammatory effects^99^. In addition, OMT can improve behavioural performance and reduce the density of Aβ plaques and astrocyte clusters in model mice^100^ (Figure 15).

Pei-Liang et al. measured the neuroprotective effect of 13 OMT derivatives on Aβ1–42-treated PC12 cells and found that compounds **68, 69,** and **70** (Figure 15) reduced more efficiently IL-1β levels and reversed cellular damage. The introduction of the benzyl group enhanced the inhibitory activity. Compound **68** exhibited the best neuroprotective effect among all derivatives, increasing cell viability by 98.37% at 1 μM and 103.13% at 5 μM. Compound **68** (5 μM) significantly reduced IL-1β levels in PC12 cells thus reducing damage caused by neuroinflammation^93^ (Figure 15, Table 5).

## Conclusion and outlook

According to the findings in the 2018 World Alzheimer’s Disease report, there is one case of AD worldwide every three seconds. The sharp increase in incidence has aroused global concern. A variety of natural products for the prevention and treatment of AD have been studied *in vivo* and *in vitro*, and many natural products can improve the pathological state of AD through multi-targets, so natural products are more suitable for multi-target drug molecular design, which is currently recognised as the most effective method. Natural products also have some defects, such as poor water solubility, high lipid dissolution, strong cytotoxicity and so on. Some shortcomings can be improved by structural modification of structure-activity relationship. For example, the introduction of amino acids, short peptides and electron-absorbing groups into natural products such as betulinic acid, 3-oxyoleanolic acid, glycyrrhizic acid and homoaconitine can greatly improve their solubility and biological activity and significantly reduce toxic reactions. However, the clinical application of natural products is limited because the active groups in the structure of some natural products cannot be confirmed. Therefore, the development of clinical drugs based on the pharmacological properties of natural products is a primary goal. This review summarises the recent five years studies on natural products and their derivatives that have been shown to ameliorate AD pathology. We hope to provide potential lead drug candidates for developing drugs against AD.
